# Chemical effects induced by the mechanical processing of granite powder

**DOI:** 10.1038/s41598-022-12962-3

**Published:** 2022-06-08

**Authors:** Anna Laura Sanna, Maria Carta, Giorgio Pia, Sebastiano Garroni, Andrea Porcheddu, Francesco Delogu

**Affiliations:** 1 Dipartimento di Ingegneria Meccanica, Chimica, e dei Materiali - CSGI Cagliari research unit, Università degli Studi di Cagliari, via Marengo 2, 09123 Cagliari, Italy; 2 Dipartimento di Chimica e Farmacia, Università degli Studi di Sassari, via Vienna 2, 07100 Sassari, Italy; 3 Dipartimento di Scienze Chimiche e Geologiche, Università degli Studi di Cagliari, Cittadella Universitaria, SS 554 bivio per Sestu, 09042 Monserrato, CA Italy

**Keywords:** Chemistry, Materials science

## Abstract

Starting from 1970s, the use of mechanical forces to induce chemical transformations has radically changed vast areas of metallurgy and materials science. More recently, mechanochemistry has expanded to core sectors of chemistry, showing the promise to deeply innovate chemical industry while enhancing its sustainability and competitiveness. We are still far, however, from unveiling the full potential of mechanical activation. This study marks a step forward in this direction focusing on the chemical effects induced on the surrounding gaseous phase by the mechanical processing of granite. We show that fracturing granite blocks in oxygen can result in the generation of ozone. The refinement of coarse granite particles and the friction between fine ones are also effective in this regard. Combining experimental evidence related to the crushing of large granite samples by uniaxial compression and the ball milling of coarse and fine granite powders, we develop a model that relates mechanochemical ozone generation to the surface area effectively affected by fracture and frictional events taking place during individual impacts. We also extend the investigation to gaseous phases involving methane, oxygen, benzene and water, revealing that chemical transformations occur as well.

## Introduction

Mechanochemistry can be broadly defined as the branch of Chemistry that studies the chemistry activated and driven by the application of mechanical forces to chemical systems including, at least, one solid phase that is deformed beyond its yield point^1–5^. At present, this field of study is experiencing a period of remarkable flourishing characterized by the exponential growth of dedicated literature and the undertaking of international actions aimed at coordinating research and innovation activities^6,7^.

The unique mix of fundamental questions and practical applications that characterizes the mechanical activation of solids has attracted enormous interest starting from 1970s, when the first generation of oxide-dispersion-strengthened superalloys were fabricated^8–11^. A second, most marked surge of interest occurred in the 1990s, when the preparation of amorphous alloys and nanostructured materials by ball milling (BM) radically changed powder metallurgy and materials science in general^12–21^.

In the recent years, the mechanical processing of powders by BM has gradually expanded to inorganic and organic synthesis, thus approaching the broadest areas of chemistry. Due to the evident promise of bringing innovation in the fine chemical industry and enhancing its sustainability, mechanochemistry has been included among the ten technological innovations that can change our world^22^. To this aim, however, a full characterization of mechanochemical reactors and processing conditions, still fragmentary, is needed^23–30^, and a deeper understanding of the chemical reactivity induced by mechanical forces must be achieved. In this regard, kinetic investigations aimed at relating microscopic processes underlying mechanochemical transformations to processing variables and conditions are key to success^31–47^.

In general, the application of a mechanical force to a solid generates mechanical stresses that, in turn, give rise to mechanical deformation. As the solid is deformed beyond its elastic limit, chemical bonds get involved in deformation and the breakage of some of them can help accommodating local strain. Breaking and reforming of chemical bonds in sequence allows the nucleation and propagation of dislocations, and dislocation slip mediates plastic deformation^48^.

Continued deformation generates new dislocations and the increase of dislocation density makes the solid increasingly harder due to dislocation interference. Progressively, the solid develops such resistance to further plastic deformation that the yield stress tends to become larger than the fracture stress. Under such conditions, cracks nucleate and grow. Eventually, the solid undergoes fracture and breaks apart in two or more fragments^48^.

The rupture of chemical bonds during dislocation motion, crack generation and growth, and fracture clearly highlights the robust bridge connecting mechanics with chemistry. However, there is much more in this respect. Together with local thermal effects, the opening of new surfaces on short time scales in ionic solids can excite electromagnetic phenomena involving the emission of neutral and charged particles and photons^3,49^. Charge separation due to fracture can generate very intense electric fields and electrons can be ejected from surface by field emission, which can also result in luminescence and electromagnetic waves^3,49^. Tribological events can give rise to similar behaviour^50^. Even at low stresses and sliding speeds, frictional electrification can produce enough electric charges to induce the dielectric breakdown of gases with consequent electric discharges and photon emission^50,51^.

The phenomena mentioned above can have immediate consequences for gaseous chemical species surrounding the deformed solid. In particular, they can induce chemical reactions in the gas phase as a result of the direct interaction between the excited solid surface and the molecules around^51^. This makes the chemical transformations in gaseous phase activated by mechanical deformation a natural subject for mechanochemistry^20^.

In this work, we provide quantitative evidence of the mechanochemical effects caused by the fragmentation and frictional processes undergone by a simple mineral such as granite. To this aim, we use BM^20,52,53^.

Much of the undeniable success of BM is due to its ease of operation. Once balls and powder have been placed inside the reactor, this is sealed under the desired atmosphere and clamped to the ball mill. Depending on the device, balls and powder are set in motion by the movement of the reactor itself or of its mobile parts. After a certain time interval, BM is stopped, the reactor opened and the powder inside sampled or retrieved.

Nevertheless, such apparent simplicity hides considerable complexity. Powder is vigorously stirred and its particle dynamics becomes more and more cooperative and dissipative as the powder amount increases. Stirring and blending are enhanced by the milling balls, which also collide with each other and with the reactor walls. The powder particles trapped between the colliding surfaces undergo non-confined dynamic compaction at relatively high strain rates. Mediated by force chains formed across the volume of trapped powder, localized severe deformation processes take place, rapidly leading to fracture in the case of brittle solids.

The processes mentioned above take place concurrently and involve a broad range of length and time scales. In spite of this, we show that detailed information on the fragmentation of brittle materials and on the chemistry activated by fragmentation and frictional events can be still obtained. In particular, we use BM experiments to evaluate the amount of coarse granite powder that can be affected by fracture during individual impacts and the related increase of specific surface area induced by fragmentation. Granite was chosen because of its effectiveness in generating chemical effects in the surrounding atmosphere^54^. In particular, in agreement with literature^54^, we observe that surfaces formed by fracture activate gaseous oxygen (O_2_), determining the formation of ozone (O_3_). We relate the degree of chemical conversion of O_2_ to O_3_ to individual impacts and provide a self-consistent kinetic description of the mechanochemical processes underlying O_3_ generation. Eventually, we extend the investigation to gaseous methane (CH_4_) and to equimolar CH_4_–O_2_ and benzene (C_6_H_6_)–water (H_2_O) gaseous mixtures, showing that chemical transformations are triggered also in these cases.

### Preliminary experiments on granite blocks under O_2_ atmosphere

Previous work has shown that the fracture of igneous and metamorphic rocks in air can produce O_3_ at ambient pressure^54^. Up to 4500 ppbv of O_3_ were instantaneously detected, and a total of 0.4 μmol were generated, from the fracture of a 30 × 30 × 15 mm^3^ granite block inside a 5-dm^3^ isolated chamber under atmospheric pressure conditions and with background O_3_ around 1.5 ppbv^54^.

**Figure 1:**
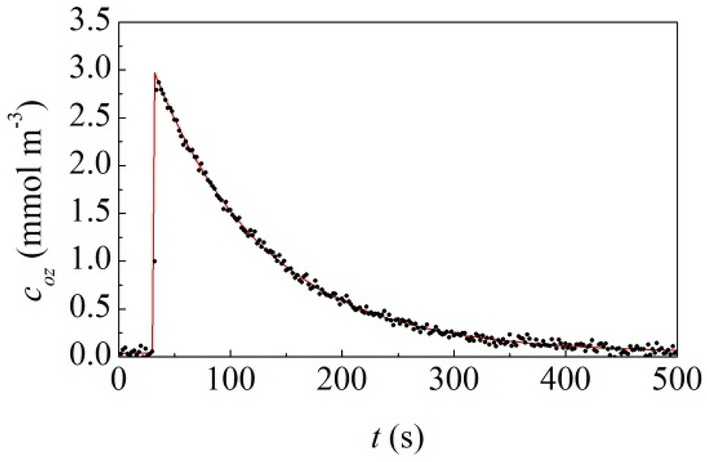
Measured O_3_ concentration, $c_{oz} \left( t \right)$, as a function of time, $t$. Data refer to experiments in which granite blocks were subjected to uniaxial compression until fracture occurred. The best-fitted curve is shown.

We carried out similar fracture experiments with 30 × 30 × 30 mm^3^ granite cubes sealed in a plastic container about 216 cm^3^ in volume under pure O_2_ gas at ambient pressure (see Supplementary Information for details). Granite blocks were subjected to uniaxial compression until fracture occurred. The atmosphere was immediately sampled and analyzed using an O_3_ monitor device. Measured O_3_ levels, $c_{oz}$, are shown in Fig. 1 as a function of time, $t$. It can be seen that O_3_ concentration suddenly rises as fracture occurs and, then, decreases with an exponential-like trend, reasonably due to ambient processes involving recombination of O atoms with either the container walls or the granite surface. After about 500 s, O_3_ concentration approximately goes back to the initial background value.

Experimental datasets can be satisfactorily best-fitted assuming that a maximum O_3_ concentration, $c_{oz,max}$, is produced instantaneously and that it decays exponentially according to the equation

1 $$ c_{oz} \left( t \right) = c_{oz,max} \exp \left( { - k_{rec} t} \right) + c_{oz,0} , $$

where $c_{oz,0}$ is the background O_3_ level and $k_{rec}$ measures the O_3_ recombination rate. In agreement with experimental measurements, the best-fitted curve suggests that, on the average, the fracture of the granite block results in a total concentration, $c_{oz,max}$, around 2.6 mmol m^−3^, which corresponds to a total of about 0.56 μmol of O_3_ generated. The background O_3_ level, $c_{oz,0}$, is around 1.8 ppbv, whereas the best-fitted $k_{rec}$ value is equal to 0.010 s^−1^.

We also evaluated the specific surface area of the granite samples before and after fragmentation (see Supplementary Information for details). The difference accounts for the surface area opened by the fracture process and is equal to about 169 cm^2^. Therefore, the amount of O_3_ generated per unit surface area generated by fracture is around 33.1 μmol m^−2^.

### Mechanical processing of granite powder under inert atmosphere

Preliminary BM experiments have been performed to monitor the gradual refinement of powder particles caused by repeated impacts. To this aim, large granite pieces were crushed manually into shards around 2 cm in size. In a first set of experiments, shards were subjected to BM for very short time intervals in order to obtain coarse granules, which were subsequently separated from finer fractions. In a second set, BM was prolonged to refine the particle size to the finest possible fraction (see Supplementary Information for details).

In all cases, BM experiments were carried out using 5 g of granite powder and a cylindrical hardened steel reactor 2.0 cm in diameter containing a single 20-g stainless steel ball approximately 1.8 cm in diameter. This means that the ball adapts almost perfectly to the 1.0-cm curvature radius of the hemispherical bottom base of the reactor (see Supplementary Information for details), which allows the powder to b effectively trapped at impact and processed. Once sealed under inert Ar atmosphere, the reactor was subjected to a vertical harmonic oscillation with amplitude and frequency suitably set to make the ball collide with the bottom of the reactor every 2 s with an impact velocity of about 2.83 m s^−1^ (see Supplementary Information for details).

**Figure 2:**
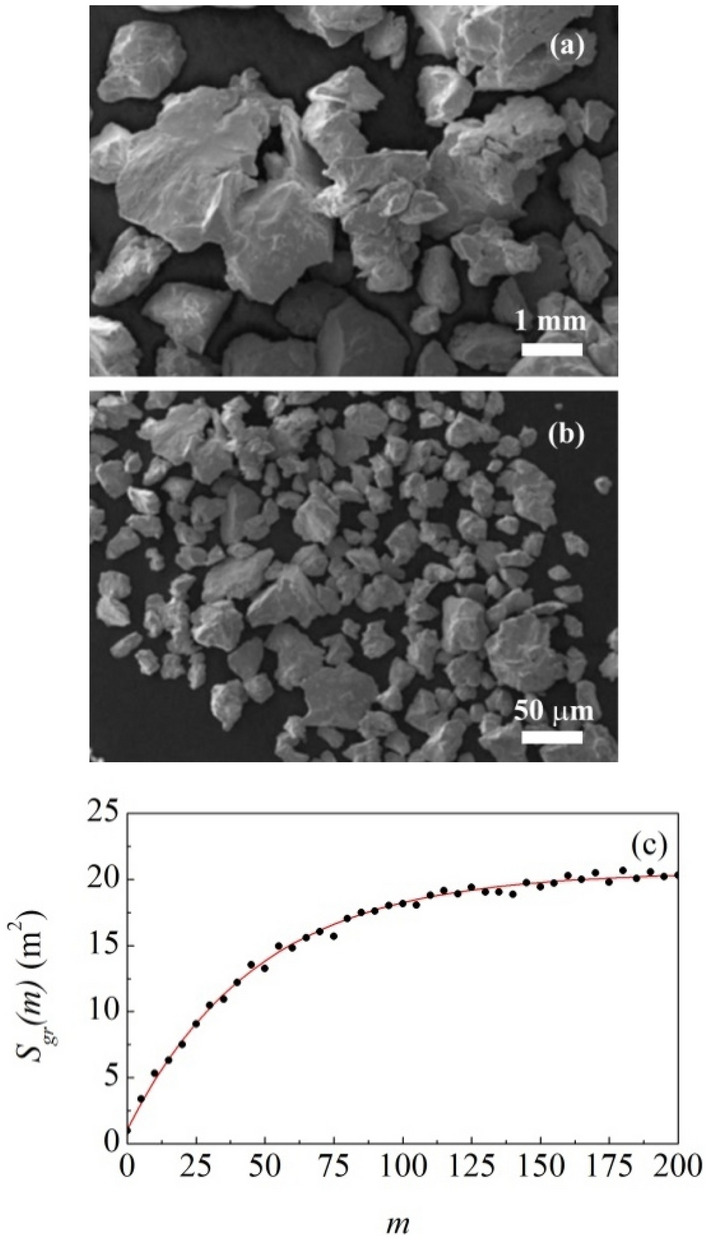
SEM micrographs of (**a**) coarse and (**b**) fine granite powder. Particle size distributions are shown. (**c**) The total surface area of granite powder, $S_{gr} \left( m \right)$, as a function of the number of impacts, $m$. The best-fitted curve is shown.

The particle size of coarser and finer powders was measured by scanning electron microscopy (SEM). Representative SEM micrographs are reported in Fig. 2a,b. It can be seen that coarse powder exhibits average particle size around 1 mm and a relatively broad size distribution, whereas the powder subjected to prolonged BM has much finer particle size and narrower size distribution (see Supporting Information for details).

SEM observation carried out on powder sampled at intermediate time intervals during long BM experiments reveals that particle size refinement takes place gradually. The process was monitored performing systematic measurements of the granite powder surface area according to the Brunauer–Emmett–Teller (BET) method (see Supporting Information for details). It can be seen from Fig. 2c that the total surface area increases progressively with the number of impacts, $m$. The observed behaviour can be satisfactorily described by the equation (see Supporting Information for details)

2 $$ S_{gr} \left( m \right) = \left( {1 - k} \right)^{m} S_{gr,in} + \left[ {1 - \left( {1 - k} \right)^{m} } \right] S_{gr,fin} $$

where $S_{gr,in}$ and $S_{gr,fin}$ are the initial and final values of surface area and $k$ is the apparent rate constant of surface area generation. According to best-fitting, the quantities $S_{gr,in}$ and $S_{gr,fin}$ have values equal to 1.10 ± 0.02 m^2^ and 20.57 ± 0.01 m^2^ respectively. The apparent rate constant, $k$, is equal to 2.1 × 10^–2^ ± 0.3 × 10^–2^. It follows that the first impact induces an increase in total surface area of about 0.41 m^2^.

The gaseous atmosphere was also sampled periodically during the mechanical processing using a gastight syringe. Through a gastight septum connector, the sampled gas was injected into a small chamber directly connected with the O_3_ monitor device. In agreement with expectations, no O_3_ was detected.

### Mechanical processing of coarse granite powder under O_2_ atmosphere

A first set of BM experiments was carried out using 5 g of coarser granite granules and the 20-g ball. The reactor was sealed under Ar atmosphere and, subsequently, Ar was replaced with O_2_ (see Supporting Information for details).

**Figure 3:**
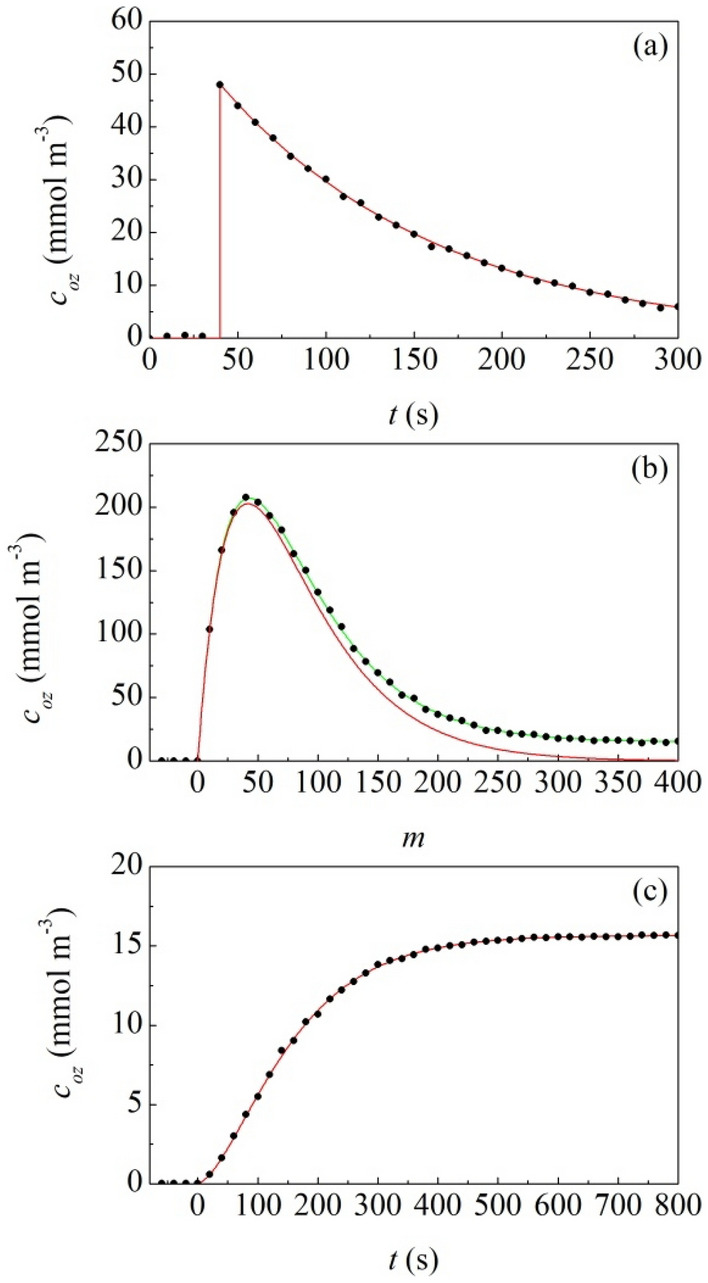
(**a**) Measured O_3_ concentration, $c_{oz} \left( t \right)$, as a function of time, $t$. Data refer to experiments in which coarse granite powder is subjected to single impacts. (**b**) Measured O_3_ concentration, $c_{oz} \left( t \right)$, as a function of the number of impacts, $m$. Data refer to experiments in which coarse granite powder is subjected to prolonged mechanical processing. (**c**) Measured O_3_ concentration, $c_{oz} \left( t \right)$, as a function of time, $t$. Data refer to experiments in which fine granite powder is subjected to prolonged mechanical processing in the absence of milling ball. Best-fitted curves are shown. In (**b**), the red curve does not account for the O_3_ generation by frictional processes involving powder particles, whereas the green curve takes into account the frictional contribution.

Initially, experiments consisted of single impacts. After the impact, the gaseous atmosphere was analyzed every 10 s in order to evaluate the amount and lifetime of O_3_ produced. Results are shown in Fig. 3a. The O_3_ monitor device indicates that O_3_ concentration increases steeply immediately after the impact, reaches a maximum and, then, decreases to the background level with an exponential-like trend. After 500 s, the O_3_ concentration decay is complete. Overall, the observed behaviour is similar to the one of O_3_ generated by fragmentation of large granite blocks. Equation 1 can be used profitably to best fit the experimental data. The maximum O_3_ concentration, $c_{oz,max}$, is around 48.1 mmol m^−3^ and the background O_3_ level, $c_{oz,0}$, is approximately equal to 4.5 ppbv. The best-fitted $k_{rec}$ value is found equal to 0.008 s^−1^.

Taking into account that the reactor has a volume of about 264 cm^3^, the total amount of O_3_ produced as a result of a single impact averages around 12.7 μmol. According to the experiments performed under Ar atmosphere, a single impact on coarse granite granules generates approximately 0.41 m^2^ of fresh surface area. Therefore, the amount of O_3_ produced per unit surface area generated by fracture during the impact is equal to about 30.9 μmol m^−2^. This value is quite close to the one of 33.1 μmol m^−2^ obtained from fracture experiments of granite blocks under O_2_ atmosphere.

We can reasonably deduce that the amount of O_3_ generated by fracture per unit surface area does not vary significantly with experimental conditions. This means that the amount of O_3_ detected after single impacts can be utilized to estimate the surface area generated by fracture and vice versa. For simplicity, we assume that the amount of O_3_ generated per unit surface area is equal to 33.1 μmol m^−2^. Hereafter, we denote it by $k_{f}$.

Prolonged BM experiments involving coarse granite powder were performed to monitor the O_3_ production by fracture on the long term. Atmosphere was sampled every 10 s and analyzed with the O_3_ monitor device. The measured O_3_ concentration is plotted in Fig. 3b as a function of the number of impacts, $m$. It can be seen that it increases smoothly, then reaches a maximum and, finally, attains a plateau value of about 15.5 mmol m^−3^.

In principle, the observed variation of O_3_ concentration can be explained taking into account three different contributions, namely the O_3_ generated by fracture, the O_3_ recombined at reactor walls or on the granite surface and the background O_3_ level. The three contributions can be readily included in a simplified modeling description of the O_3_ concentration variation that takes into due account the discrete nature of BM and, then, of O_3_ production (see Supplementary Information for details). Specifically, the O_3_ generated in a given impact is assumed proportional to the change in granite surface area caused by fragmentation during such impact and the decay process of the O_3_ amount generated is assumed to start immediately after the impact. The result is the expression

3 $$ c_{oz,f} \left( m \right) = k_{f} k \left( {S_{gr,fin} - S_{gr,in} } \right)\exp \left[ { - k_{rec} \left( {m - 1} \right) \tau } \right]\frac{{1 - \left[ {\left( {1 - k} \right)\exp \left( {k_{rec} \tau } \right)} \right]^{m} }}{{1 - \left( {1 - k} \right)\exp \left( {k_{rec} \tau } \right)}} + c_{oz,0} $$

where $k_{rec}$ is the rate constant for O_3_ recombination and $\tau$ is the time interval separating two consecutive impacts.

Equation 3 relates the O_3_ generation to individual impacts. In principle, it can be used to predict the variation of $c_{oz}$ as the number of impacts increases. The time interval $\tau$ between consecutive impacts is known and $k_{f}$, $S_{gr,in}$, $S_{gr,fin}$, $k$, $k_{rec}$ and $c_{oz,0}$ have been evaluated in previous experiments. However, we cannot exclude that the rate of recombination process, $k_{rec}$, changes in the presence of repeated impacts and powder stirring. For this reason, we use Eq. (3) to best-fit the experimental points leaving $k_{rec}$ free to vary as the only best-fitting parameter.

As shown in Fig. 3b, Eq. (3) is able to capture the general trend of experimental data. With a best-fitted $k_{rec}$ value of about 0.014 s^−1^, it well reproduces the initial increase, the maximum and the subsequent exponential decay. However, it is definitely unable to match the final plateau value.

In this respect, it is worth noting that the final values attained by O_3_ concentration are much higher than the expected background level, $c_{oz,0}$, much lower than 15.5 mmol m^−3^. The solution to this apparent inconsistency comes from the experiments performed on finer granite powder.

Experiments were carried out, both in the presence and in the absence of the milling ball, under O_2_ atmosphere for long mechanical processing times. The reactor atmosphere was analyzed every 10 s with the O_3_ monitor device. The O_3_ concentration measured during experiments carried out in the absence of milling ball is shown in Fig. 3c as a function of time. Experimental data reveal that O_3_ concentration increases smoothly, reaching a plateau value of about 15.3 mmol m^−3^.

This value is significantly higher than the background O_3_ level observed in experiments involving single impacts and substantially coincident with the final plateau value observed in experiments involving the prolonged mechanical treatment of coarser granite powder. Approximately the same behaviour is observed both in the presence and in the absence of the milling ball. We infer that O_3_ generation takes place despite no significant fracture process occurs to modify the particle size of granite power. Therefore, we ascribe the observed O_3_ generation to frictional processes involving the surface of powder particles.

Based on such hypothesis, a suitable modeling description can be developed (see Supporting Information for details). Experimental data can be satisfactorily best-fitted by the expression

4 $$ c_{oz,a} \left( m \right) = k_{a} \exp \left[ { - k_{rec} \left( {m - 1} \right) \tau } \right]\left[ {S_{gr,fin} \frac{{1 - \left[ {\exp \left( {k_{rec} \tau } \right)} \right]^{m} }}{{1 - \exp \left( {k_{rec} \tau } \right)}} - \left( {S_{gr,fin} - S_{gr,in} } \right)\left( {1 - k} \right)\frac{{1 - \left[ {\left( {1 - k} \right)\exp \left( {k_{rec} \tau } \right)} \right]^{m} }}{{1 - \left( {1 - k} \right)\exp \left( {k_{rec} \tau } \right)}}} \right] + c_{oz,0} , $$

which allows estimating the amount of O_3_ generated by attrition per unit surface area, $k_{a}$. The best-fitted $k_{a}$ estimate is equal to about 0.02 μmol m^−2^. Therefore, it is three orders of magnitude smaller than the amount of O_3_ generated by fracture surfaces, $k_{f}$, which has been found equal to about 33.1 μmol m^−2^.

If we assume that friction between particles generates O_3_ also during the mechanical processing of coarse granite powder, Eq. (3) has to be re-written as (see Supplementary Information for details)

5 $$ c_{oz} \left( m \right) = c_{oz,f} \left( m \right) + c_{oz,a} \left( m \right). $$

In fact, Eq. (5) best fits the experimental data in Fig. 3b with a background O_3_ level, $c_{oz,0}$, of about 5.3 ppbv.

The estimated values of the different quantities are summarized in Table 1. It can be seen that there is substantial agreement between the values obtained in different experiments. It follows that systematic experiments and modeling description have been able to unveil parts of the more complex process of mechanical processing by BM. Under these circumstances, the model can, in principle, give rise to reliable predictions.

**Table 1:** Parameter values used to best-fit the experimental data obtained in different experiments.

	$c_{oz,0}$	$c_{oz,max}$	$k_{f}$	$k_{a}$	$k_{rec}$
Compression	0.04	2.96	33.1		0.010
Single impact	0.06	12.4	30.9		0.008
Milling	0.05		33.1	0.02	0.014
Stirring	0.05			0.02	0.014

The model parameters considered are background O_3_ level, $c_{oz,0}$ (mmol m^−3^), the maximum O_3_ concentration measured after fracture processes induced by uniaxial compression of granite cubes and individual impacts on granite coarse powder, $c_{oz,max}$ (mmol m^−3^), the amount of O_3_ generated by fracture per unit surface area, $k_{f}$ (μmol m^−2^), the amount of O_3_ generated by attrition per unit surface area, $k_{a}$ (μmol m^−2^) and the O_3_ recombination rate, $k_{rec}$ (s^−1^).

### Mechanical processing of granite powder under CH_4_ and equimolar CH_4_–O_2_ atmospheres

If electromagnetic phenomena are involved in O_3_ generation, the formation of new surfaces by fracture or friction between powder particles can be expected to drive to reaction chemical species different from O_2_. To investigate this issue, we focused on the chemical response of gaseous CH_4_. This choice is related to the availability of literature on the reactivity of gaseous CH_4_, and organic compounds in general, in the presence of glow discharges^55,56^.

Experiments were performed under the same processing conditions utilized for experiments under O_2_ atmosphere. Specifically, about 5 g of coarse granite granules were introduced in the reactor together with the 20-g ball. Then, the reactor was sealed under Ar atmosphere and Ar suitably replaced with CH_4_. The gaseous atmosphere was sampled and analyzed by gas-chromatography using a flame ionization detector (see Supporting Information for details).

**Figure 4:**
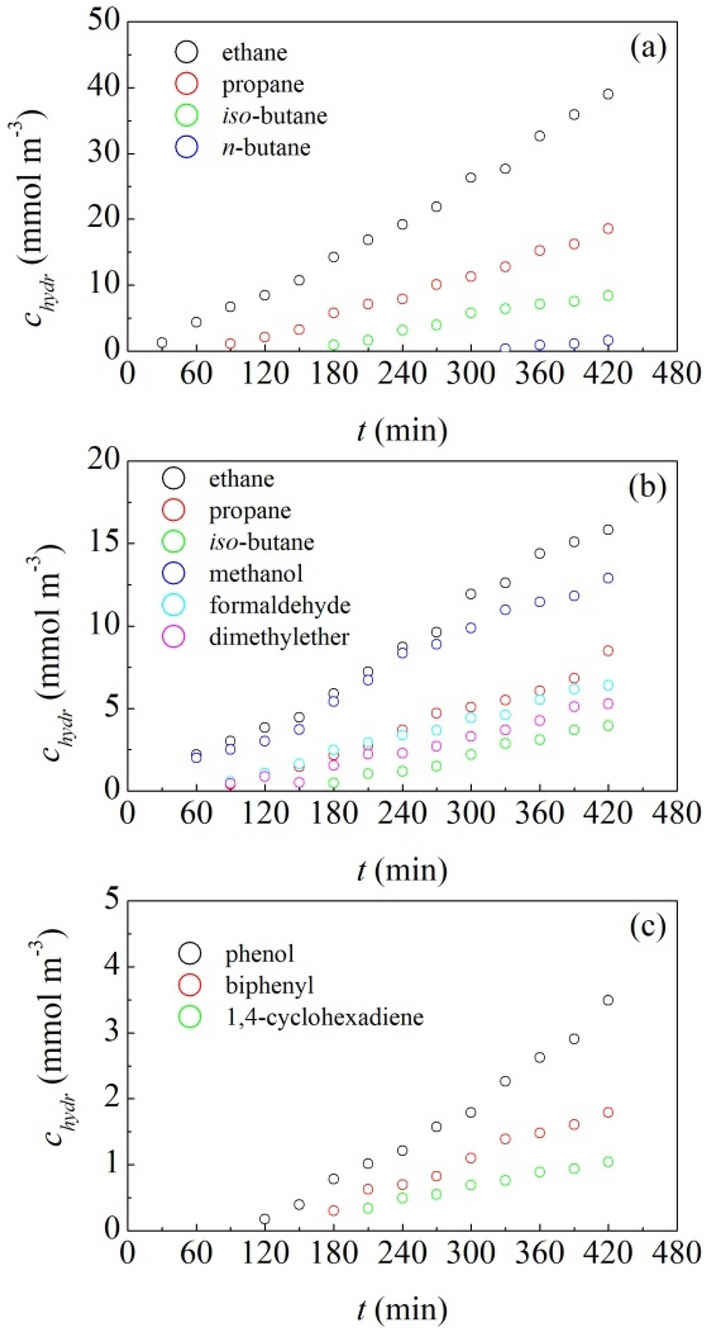
Measured concentration of chemical species, $c_{hydr} \left( t \right)$, as a function of time, $t$. Data refer to experiments in which coarse granite powder is subjected to prolonged mechanical processing in the presence of (**a**) gaseous CH_4_, (**b**) an equimolar CH_4_–O_2_ gaseous mixture, and (**c**) an equimolar C_6_H_6_–H_2_O gaseous mixture. The chemical species formed are indicated in the plots.

Experimental data shown in Fig. 4a clearly reveal the partial conversion of CH_4_ to ethane (CH_3_CH_3_) and other light hydrocarbons. Therefore, the prolonged mechanical processing of granite by BM activates CH_4_ molecules and allows their combination in more complex molecular units.

No kinetic modeling based exclusively on mechanochemical processes occurring during individual impacts can be developed in the present case. Indeed, the mechanochemical kinetics responsible for the generation of the chemical species that eventually allow the formation of hydrocarbons is inextricably intertwined with chemical kinetics in the gaseous phase. Therefore, no direct relationship can be expected between the concentration of gaseous species and the fragmentation and frictional processes undergone by granite.

Similar considerations hold for the experiments performed using an equimolar mixture of CH_4_ and O_2_ gases. In this case, gas-chromatographic analyses allow detecting CH_3_CH_3_ and light hydrocarbons as well as oxidized chemical species such as methanol (CH_3_OH), dimethyl ether (CH_3_OCH_3_) and formaldehyde (CH_2_O). The concentrations of a few chemical species are shown in Fig. 4b as a function of time. Chemical species appear, more or less, in order of increasing complexity, suggesting that simpler compounds combine with each other to form more complex ones. The concentration of the different chemical species increases as time increases.

### Mechanical processing of granite powder under equimolar C_6_H_6_–H_2_O atmosphere

In the attempt to further explore the chemistry of gaseous phases initiated by the mechanical processing of granite, we carried out prolonged BM experiments on granite powder in the presence of gaseous C_6_H_6_ and H_2_O. To this aim, experiments were performed at 150 °C using the same processing conditions utilized for other atmospheres (see Supporting Information for details).

As shown in Fig. 4c, we observe the conversion of C_6_H_6_ to different complex species. Those with higher concentration are biphenyl (C_12_H_10_), phenol (C_6_H_5_OH) and 1,4-cyclohexadiene (C_6_H_8_). Again, the prolonged mechanical processing of granite shows the capability of inducing chemical reactivity in organic molecules.

## Discussion

The mechanical processing of granite powder gives rise to chemical effects in the presence of reactive gases. Literature suggests that such effects can be ascribed to the electrons emitted as a consequence of the intense electric fields that are generated when a fracture separates electrical charges located on the parting surfaces^54^. The heterolytic rupture of chemical bonds, and the consequent formation of ions, can result in electric fields of about 1.8 × 10^–6^ $\rho$ V cm^−1^^9^, where $\rho$ is the surface density of separating charges. The electric breakdown of air takes place at electric fields around 30 kV cm^−1^, which require $\rho$ values approximately equal to 1.7 × 10^10^ cm^−2^. The surface density of ions needed to activate discharges in gaseous O_2_ and CH_4_ is on the same order of magnitude, i.e. about 1.4 × 10^10^ cm^−2^ and 2.9 × 10^10^ cm^−2^ respectively. These $\rho$ values can be compared with the surface density of ions in surfaces freshly generated by fracture, which can be as high as 1.0 × 10^15^ cm^−2^.

In this respect, it is interesting to note that the amount of O_3_ generated by fracture per unit surface area, $k_{f}$, is approximately equal to 33.1 μmol m^−2^. Under the hypothesis that the number of O_3_ molecules generated equals the number of active sites responsible for electromagnetic phenomena, such estimate indicates that we can expect a surface density of active sites of about 1.9 × 10^19^ cm^−2^. This value is definitely higher than the surface density of separating charges, $\rho$. The amount of O_3_ generated by friction per unit surface area, $k_{a}$, is approximately equal to 0.02 μmol m^−2^ and leads to a lower surface density of active sites, around 1.2 × 10^16^ cm^−2^, which is however higher than $\rho$. It follows that both fracture and frictional processes can give rise to electric fields with intensity well beyond the threshold required to activate plasma discharges in the investigated gaseous phases. This means that electromagnetic phenomena activated by fracture and friction can explain, in principle, the observed O_3_ generation and the chemical reactivity in the presence of CH_4_ as well as of CH_4_—O_2_ and C_6_H_6_—H_2_O equimolar mixtures.

In this regard, however, at least another mechanism can be invoked. Mechanical processing by BM has been shown to generate excited chemical bonds at the surface of solids that, in turn, determine the consumption of a radical scavenger^57^. Specifically, the fracture and friction of quartz particles dispersed in liquid ethanol has been shown to generate active sites with surface density respectively equal to about 4.8 × 10^13^ and 2.8 × 10^12^ cm^−2^. The surface density of active sites formed by fracture is comparable, and possibly even higher, than the maximum possible surface density of dangling bonds, which is equal to about 1.2 × 10^13^ cm^−2^ for quartz. This suggested a very high reactivity for quartz surfaces excited by mechanical activation, eventually resulting in the formation of radicals. Similar results have been obtained for pozzolanas in the presence of H_2_O^58^.

The formation of radical species can explain the reactions involving CH_4_. In particular, we can hypothesize the formation of CH_3_‧ radicals as a consequence of interaction of CH_4_ molecules with the excited granite surface. We can also expect the formation of O‧ radicals in the presence of O_2_ molecules. Therefore, the organic molecules formed in the presence of CH_4_ and O_2_ can be the result of radical reactions.

In contrast, the formation of C_6_H_8_ in the presence of C_6_H_6_ and H_2_O can be hardly explained in terms of a radical transformation. Rather, it is reminiscent of the old-fashioned Birch reduction, a widely used reaction in synthetic organic chemistry that converts benzenoid compounds into 1,4-cyclohexadienes^59^. Although reaction conditions are quite different from conventional ones using ammonia and alkaline metals^59,60^, we can still conjecture that the initial step involves the reduction of the aromatic ring by an electron. We simply remark that, in our case, electrons can be made available by electrification phenomena caused by fracture or attrition.

## Conclusions

Experimental findings prove that the mechanical processing of granite can have significant chemical effects on the surrounding gaseous phase. In the presence of oxygen, fracturing and frictional processes determine the formation of relatively small amounts of ozone. The direct relationship between ozone generation and mechanical activation allows developing an accurate kinetic model that connects the kinetic evidence on the macroscopic scale with the number of impacts, the total surface area involved in fracturing and frictional events, and the characteristic ozone lifetime under the processing conditions investigated. The kinetic model bridges successfully the global and local scales of the mechanochemical transformation, showing that experimental kinetics can be exploited to obtain information on the local chemistry underlying mechanically activated reactions. It is precisely such local chemistry that most genuinely points to the heart of the chemistry induced by the application of mechanical forces.

The kinetic model can be extended to other mechanochemical transformations characterized by a direct relationship between mechanical activation and chemical effect. This is not the case of the mechanochemical reactions induced in the presence of gases more complex than oxygen. Indeed, the global kinetics also depends, in such cases, on the intrinsic gaseous phase behaviour. And yet, it is worth noting that the mechanical processing of a simple, apparently inert mineral, and presumably of other minerals and ionic solids, can give rise to significant chemistry. We ascribe the observed mechanically induced reactivity to the generation of intense electromagnetic fields. Seemingly, this is the most reasonable working hypothesis. However, we cannot hide the fact that investigation in this area is just beginning and we have yet to face the most important challenges.

## Supplementary Information

Supplementary Information.

## Data Availability

Datasets generated and analysed during the current study cannot be shared at this time as they also forms part of an ongoing study. However, they can be available totally or in part from the corresponding author on reasonable request.
