# Supplementary material for: Chemical effects induced by the mechanical processing of granite powder
Source: Sci Rep. 2022 Jun 8;12:9445. doi: 10.1038/s41598-022-12962-3 (PMC9177845; doi:10.1038/s41598-022-12962-3)
Supplement: Supplementary file 1 — Supplementary Information. [file 41598_2022_12962_MOESM1_ESM.docx]

Chemical effects induced by the mechanical processing of granite powder

Anna Laura Sanna^1^, Maria Carta^1^, Giorgio Pia^1,2^, Sebastiano Garroni^3^, Andrea Porcheddu^4^ and Francesco Delogu^1,2*^

^1^ Dipartimento di Ingegneria Meccanica, Chimica, e dei Materiali, Università degli Studi di Cagliari, via Marengo 2, 09123 Cagliari, Italy

^2^ Center for Colloid and Surface Science (CSGI), Department of Chemistry, University of Florence, via della Lastruccia 3, 50019 - Sesto Fiorentino (FI), Italy - Cagliari research unit, via Marengo 2, 09123 Cagliari, Italy

^3^ Dipartimento di Chimica e Farmacia, Università degli Studi di Sassari, via Vienna 2, 07100 Sassari, Italy

^4^ Dipartimento di Scienze Chimiche e Geologiche, Università degli Studi di Cagliari, Cittadella Universitaria, SS 554 bivio per Sestu, 09042, Monserrato (CA), Italy

Supplementary Information

SI1. Materials

Granite rock originates from a Sardinian quarries of so-called white granite located in the municipality of Buddusò, in the province of Sassari, Italy. Rocks were worked in the shape of 30×30×30 mm^3^ cubes such as the one shown in Fig. SI1.1.


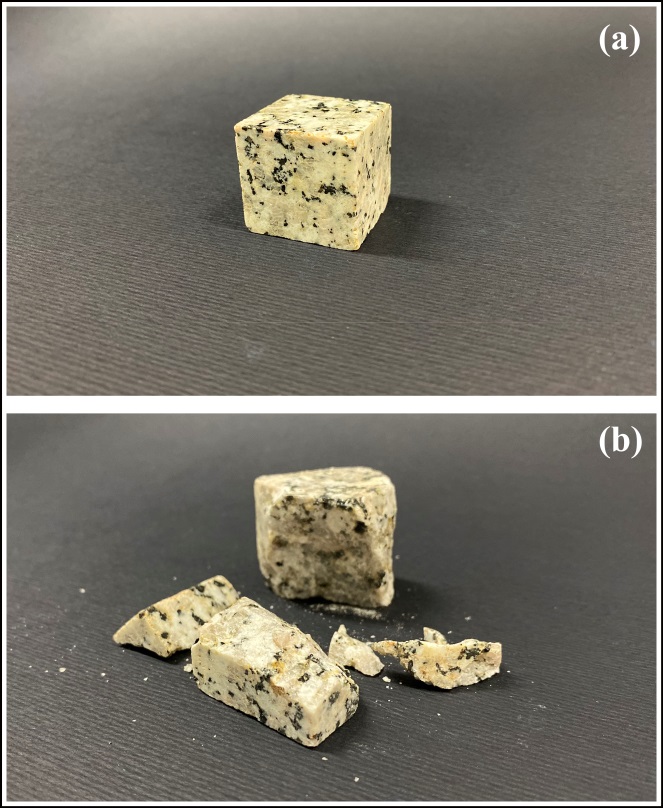


Fig. SI1.1. A granite cube used in uniaxial compression experiments.

Fragments of granite were crushed using a ball mill SPEX Mixer/Mill 8000 and transformed into fine powder to carry out suitable X-ray diffraction analysis. The obtained pattern is shown in Fig. SI1.2.


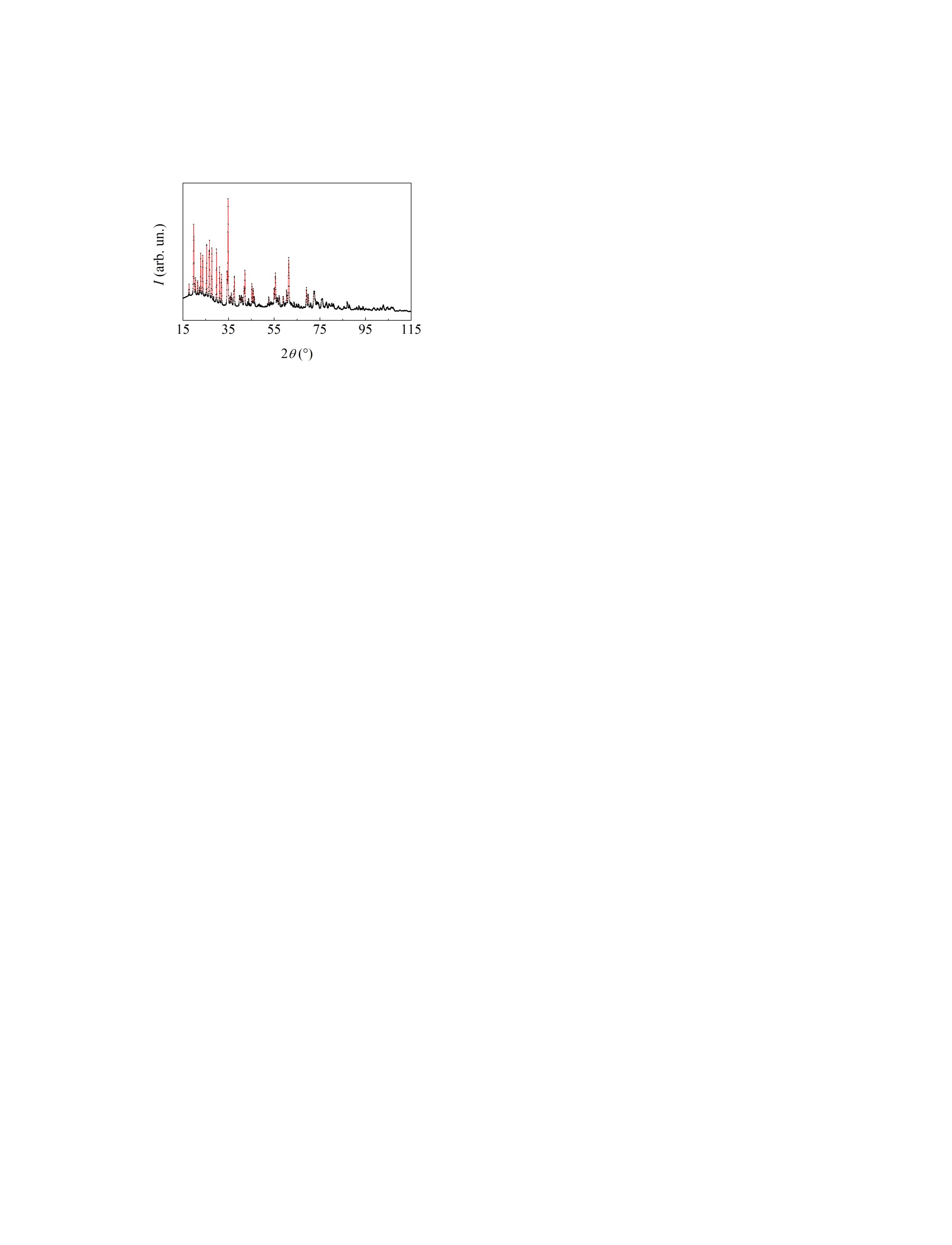


Fig. SI1.2. X-ray diffraction pattern of fine granite powders.

It appears that the granite is highly crystalline, exhibiting structure and microstructure similar to those of other granites from Sardinia (Italy) and foreign sites.

Large granite pieces formed by fracture were crushed manually into shards around 2 cm in size. Pictures are shown in Fig. SI1.3a for illustration purposes.


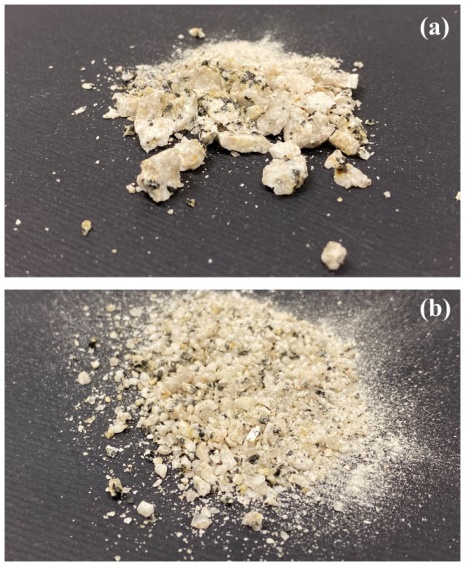


Fig. SI1.3. (a) A mixture of shards and coarse powder. (b) A mixture of coarse and fine powders.

Shards were subjected to mechanical processing for short time intervals in order to obtain coarse granules such as the ones shown in Fig. SI1.3b. Further mechanical processing allowed obtaining fine powder.

High-purity gaseous phases and chemicals were purchased from SIGMA Aldrich and used as supplied.

SI2. Uniaxial compression

Experiments were performed using an electro-hydraulic pressing machine with digital control. The motorized punch is shown in Fig. SI2.1a.


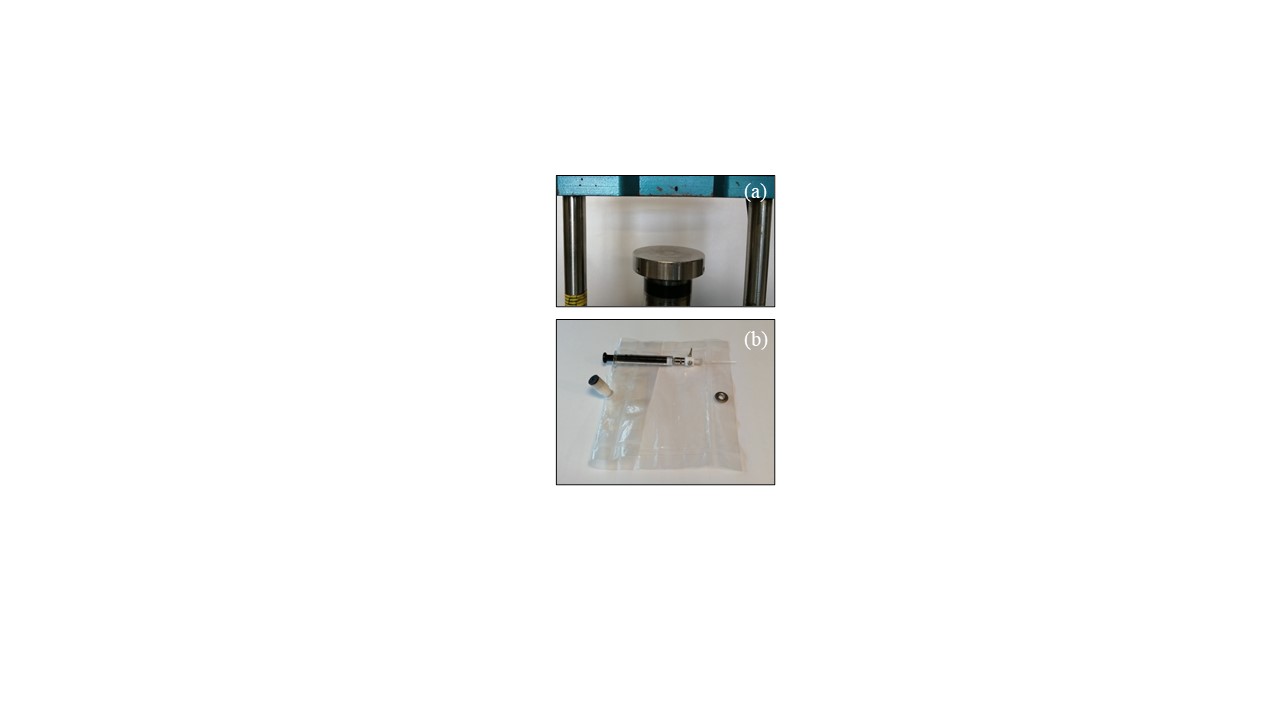


Fig. SI2.1. (a) Detail of the electro-hydraulic press utilized in uniaxial compression tests. (b) The plastic container used to isolate the granite block and sample the atmosphere.

A plastic container, such as the one shown in Fig. SI2.1b, was used to isolate the granite block from the environment. The granite block was inserted into the container and the container sealed in air using a hot metal press heated by electrical resistances. The plastic container is equipped with a gastight septum connector that allows sampling the atmosphere with a syringe or the direct connection with the ozone monitoring device.

SI3. Mechanical processing

Mechanical processing of granite powder was performed in a stainless steel mechanochemical reactor of cylindrical shape. About 2 g of granite power were placed into the reactor chamber together with a 20-g stainless steel ball. The reactor was sealed under Ar atmosphere. Atmosphere can be replaced and sampled using the gas-tight septum connector equipping the reactor screw cap.

Once sealed, the reactor was clamped to a mechanical arm that undergoes a vertical harmonic oscillation. Mechanical device and reactor are schematically depicted in Fig. SI3.1.


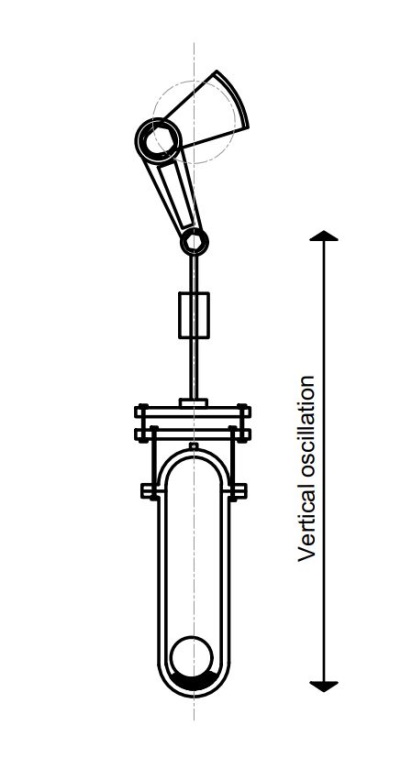


Fig. SI3.1. Schematic description of the mechanochemical reactor with gas-tight septum connector on the screw cap and the crank mechanism of the vertical vibration mill.

Under operational conditions, the single ball collides with the reactor base, thus trapping and loading part of the powder charge. Frequency and energy of impacts can be adjusted varying the amplitude and frequency of reactor oscillation. We selected processing conditions allowing impacts every 2 s. Powder charge is large enough to establish inelastic impact conditions, which result in an impact energy of about 0.07 J.

Control experiments aimed at evaluating the effect of stainless steel on the mechanochemical behaviour have been also performed using a Plexiglas reactor of the same shape and size as well as balls of tungsten carbide and zirconia. No qualitative difference was observed.

SI4. Surface area measurements

Samples in the form of coarse or fine powders were kept for 2 h at 393 K to dry. Then, the samples were subjected to degassing at 300 K and exposed to N_2_ at about 77 K in a Fisons Sorptomatic 1900 apparatus. The specific surface area was estimated by physical adsorption of nitrogen according to the so-called BET method. For each sample, measurements were repeated three times.

SI5. Electron microscopy observations

Scanning electron microscopy was performed using a Zeiss EVO LS15 microscope. Observations were carried out to determine the average size and a consequent, indirect estimate of the surface area. In each case, ten to fifteen samples of ground granite powders were investigated with the aim of obtaining reliable datasets to monitor the size refinement of granite particles. About 100 particles per sample were considered. Thus, the total number of size measurements ranged between 1000 and 1500. It is large enough to assure a satisfactory statistics.


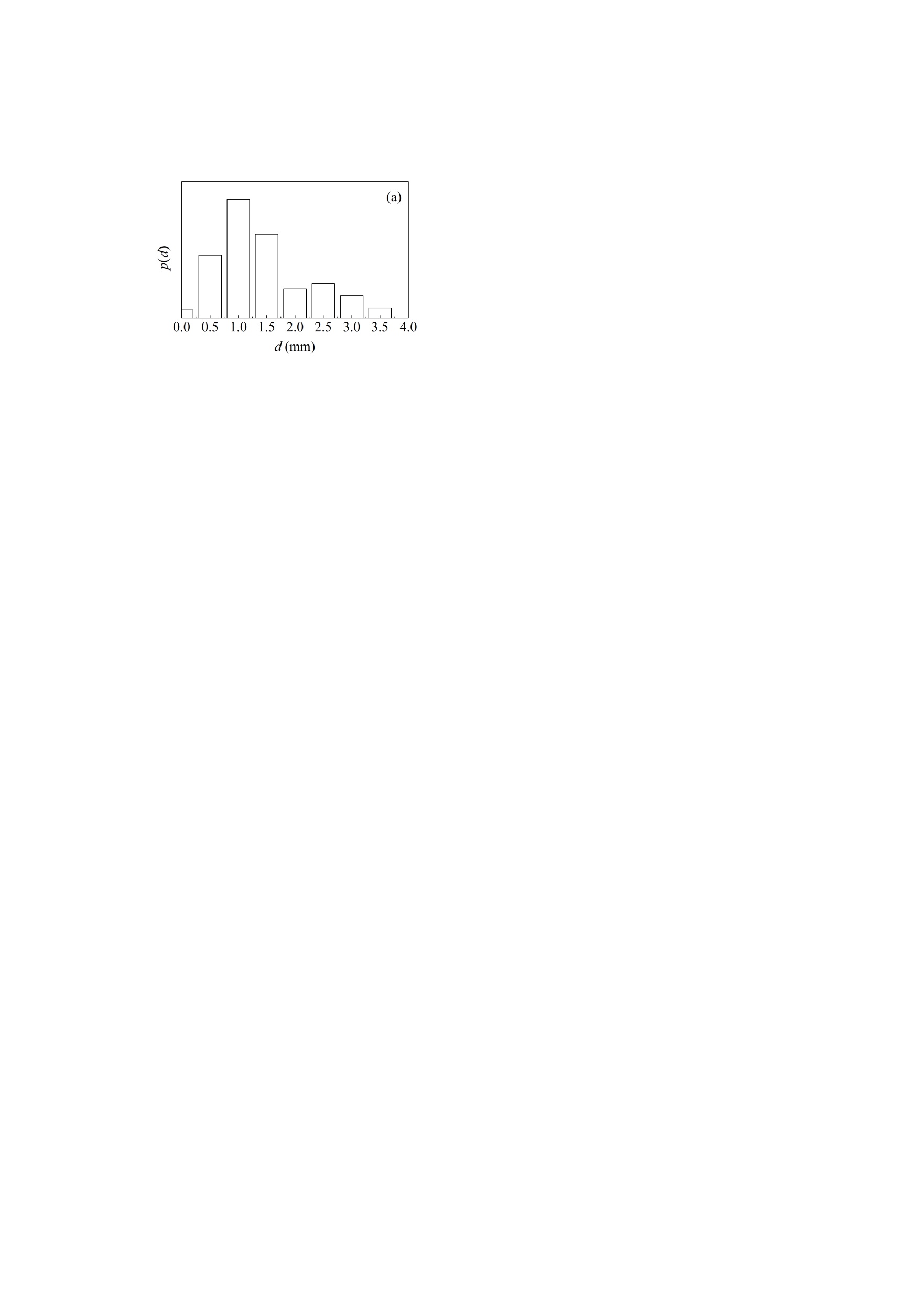


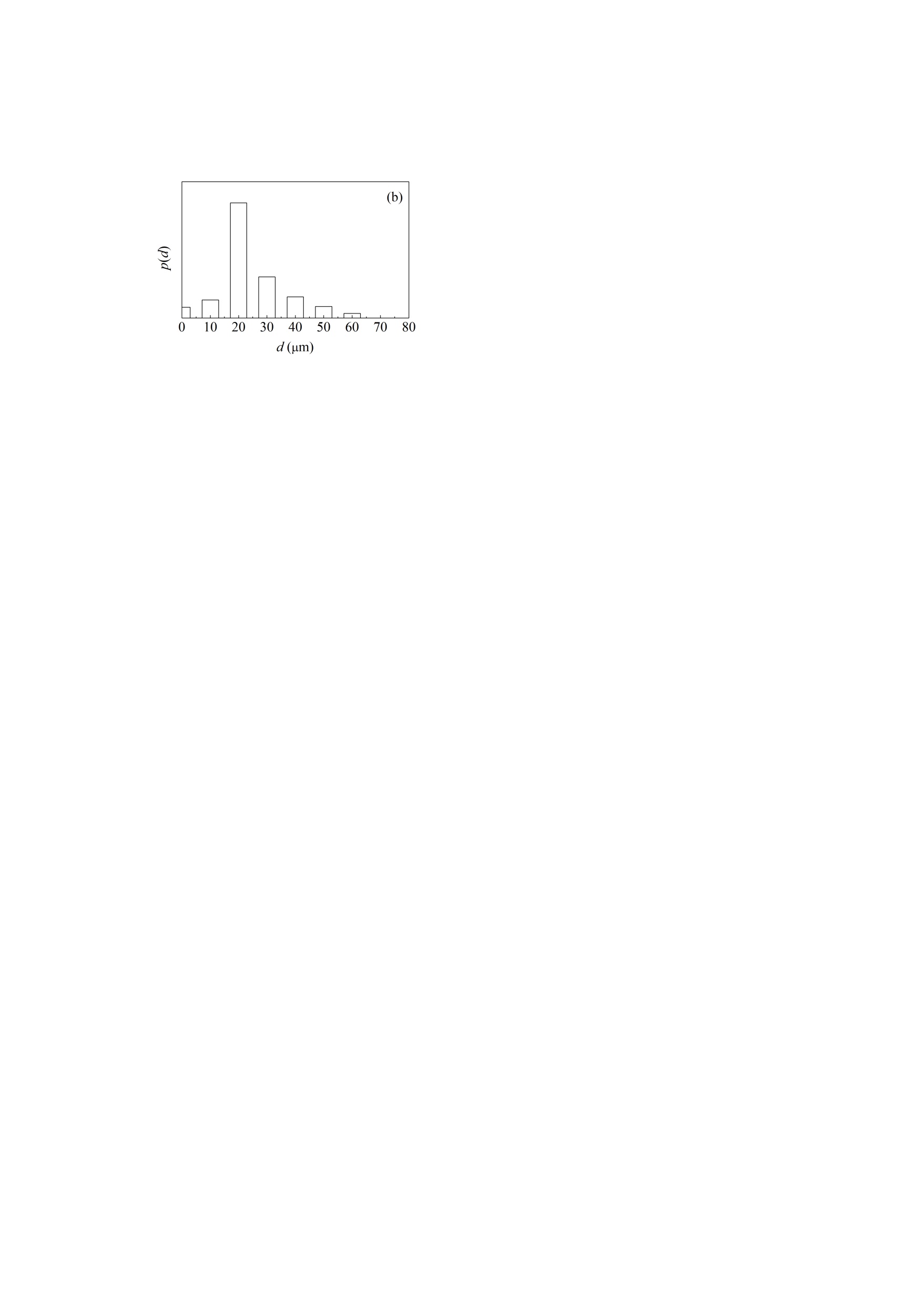


Fig. SI5.1. Statistical distributions, *p*(*d*), of the particle size, *d*, obtained from SEM image analysis of (a) coarse and (b) fine granite powder.

SI6. Gas-chromatographic analysis

A Perkin Elmer 8600 gas-chromatograph equipped with equipped with a GSQ 115- 3432-J&W Scientific capillary column and a FID detector was used to detect CH_4_ and light hydrocarbons. A Fisons 8000 gas-chromatograph equipped with a molecular sieves column (10 Å) and an HWD detector was utilized to evaluate H_2_ and CO.

Measurements were carried out sampling the gas inside the milling vial with a gastight syringe using the gas-tight septum placed on the cap of the stainless-steel vial. The sampled gas was injected into the gas-chromatographic column through the sample chamber.

Gaseous species were identified based on the comparison between the experimental retention times and the characteristic retention times of commercial standards with experimental ones. Specific standards for the quantitative analysis of the different chemical systems were provided by Gruppo Sapio (Italy). Absolute and relative amounts were determined by peak-area evaluation.

SI7. Ozone level measurements

A 2B Technologies Model 205 Dual Beam Ozone Monitor was used to measure O_3_ concentration in atmosphere. Measurements were performed in two different modes. On the one hand, the device was directly connected with the plastic container that contained the granite blocks in fracture experiments or to the reactor used to subject granite powder to mechanical processing. On the other, a gas-tight syringe was used to sample the atmosphere of the plastic container or of the reactor and to inject it into the ozone monitor device. Satisfactory agreement was always observed between measurements performed with the two methods.

Typically, data were sampled every 2 s during short experiments involving the fracture of a granite blocks or single impacts inside the reactor. During longer experiments involving the mechanical processing of coarse and fine granite powders, a sampling time of 10 s was used. In any case, experiments were repeated at least three times to achieve the desired accuracy degree.

Since the 10-s sampling time allows better accuracy, measurements regarding short experiments were occasionally repeated with a 10-s sampling time to verify the reliability of measurements performed with 2-s sampling time. Satisfactory agreement was always observed.

SI8. Kinetic model

The mechanical processing of granite powder by ball milling can be described based on a few fundamental features. First, only a small amount of granite powder is effectively processed during each impact. Second, during each impact the ball and reactor surfaces trap the powder according to an approximately stochastic dynamics. Finally, the amount of processed powder remains approximately constant during the entire mechanical processing.

Let us define $\boldsymbol{k}$ as the volume fraction of powder effectively processed during each impact. The powder charge can be divided into $\boldsymbol{k}^{\boldsymbol{-1}}$ equal volume elements that have the same probability of being processed in a given impact. At the first impact, the volume fraction of powder remaining unaffected is

$\chi_{0}\left( 1 \right)=1-k$. (SI8.1)

After two impacts, it becomes

$\chi_{0}\left( 2 \right)=1-k-k\left( 1-k \right)=\left( 1-k \right)^{2}$. (SI8.2)

After three impacts, it is

$\chi_{0}\left( 3 \right)=1-k-k\left( 1-k \right)-k\left[ 1-k-k\left( 1-k \right) \right]=\left( 1-k \right)^{3}$. (SI8.3)

After $n$ impacts, the volume fraction of unaffected powder is

$\chi_{0}\left( n \right)=\left( 1-k \right)^{n}$. (SI8.4)

Accordingly, the volume fraction of powder affected for the first time by the first impact is

${\Delta\chi}_{1}\left( 1 \right)=k$. (SI8.5)

The volume fraction of powder affected for the first time by the second impact is

${\Delta\chi}_{1}\left( 2 \right)=k\left( 1-k \right)$. (SI8.6)

The volume fraction of powder affected for the first time by the third impact is

${\Delta\chi}_{1}\left( 3 \right)=k\left[ 1-k-k\left( 1-k \right) \right]={k\left( 1-k \right)}^{2}$. (SI8.7)

Therefore, the volume fraction of powder affected for the first time by the $n$-th impact is

${\Delta\chi}_{1}\left( n \right)={k\left( 1-k \right)}^{n-1}$. (SI8.8)

Every time a given volume fraction of powder is affected for the first time by an impact, we assume that its surface area changes from $S_{gr,in}$ to $S_{gr,fin}$. Thus, it undergoes a surface area change of $S_{gr,fin}-S_{gr,in}$. Therefore, the total surface area changes with the number $m$ of impacts as

$\Delta S_{tot}\left( m \right)=\left( S_{granite,fin}-S_{granite,in} \right)\sum_{n=1}^{m} k\left( 1-k \right)^{n-1}=k \Delta S_{0} \sum_{n=1}^{m} \left( 1-k \right)^{n-1}$ (SI8.9)

We assume that the amount of O_3_ generated by fracture during each impact is proportional to the surface area change induced by the impact. Therefore, the amount of O_3_ generated by fracture during the first impact is equal to $k_{f} k \Delta S_{0}$, where $k \Delta S_{0}$ is the surface area change caused by the effective processing of the volume fraction of powder $k$. The total amount of O_3_ generated by fracture after $m$ impacts is

$c_{ozone}\left( m \right)=k_{f} k \Delta S_{0} \sum_{n=1}^{m} \left( 1-k \right)^{n-1}=k_{f} k \left( 1-k \right)^{-1} \Delta S_{0} \sum_{n=1}^{m} \left( 1-k \right)^{n}$. (SI8.10)

Taking into account that the sum of powers defines a geometric series, it can be re-written as

$c_{ozone}\left( m \right)=k_{f} \Delta S_{0} \left[ 1-\left( 1-k \right)^{m} \right]$. (SI8.11)

Eq. SI8.11 accounts for the total amount of O_3_ generated by fracture in the absence of recombination processes that reduce O_3_ concentration starting, for any given amount of O_3_ generated during a given impact, immediately after the impact. However, we know that O_3_ generated by fracture starts immediately decaying. Therefore, we can suppose that the amount of O_3_ generated by fracture during the first impact decreases with time according to the expression

$c_{ozone}\left( 1 \right)=k_{f} \Delta S_{0} k\exp\left[ -k_{rec} t \right]$, (SI8.12)

where $k_{rec}$ is the characteristic recombination rate constant. If $k_{rec}$ is large enough, recombination processes take place on time intervals that involve several consecutive impacts. Under such circumstances, time can be written as

$t=n \tau$, (SI8.13)

where $\tau$ is the time interval between consecutive impacts. Then, the total amount of O_3_ immediately after the second impact is

$c_{ozone}\left( 2 \right)=k_{f} \Delta S_{0} k\exp\left[ -k_{rec} \tau\right]+k_{f} \Delta S_{0} k\left( 1-k \right)$. (SI8.14)

Immediately after the third impact,

$c_{ozone}\left( 3 \right)=k_{f} \Delta S_{0} k\exp\left[ -k_{rec}2 \tau\right]+k_{f} \Delta S_{0} k\left( 1-k \right)\exp\left[ -k_{rec} \tau\right]+k_{f} \Delta S_{0} k \left( 1-k \right)^{2}$ (SI8.15)

In general, the total amount of O_3_ immediately after the $m$-th impact is

$c_{ozone}\left( m \right)=k_{f} k \left( 1-k \right)^{-1} \Delta S_{0} \sum_{n=1}^{m} \left( 1-k \right)^{n}\exp\left[ -k_{rec}\left( m-n \right) \tau\right]$. (SI8.16)

Eq. SI8.16 can be rewritten as

$c_{ozone}\left( m \right)=k_{f} k \left( 1-k \right)^{-1} \Delta S_{0}\exp\left[ -k_{rec}m \tau\right] \sum_{n=1}^{m} \left[ \left( 1-k \right)\exp\left( k_{rec}\tau\right) \right]^{n}$ (SI8.17)

and, finally, as

$c_{ozone}\left( m \right)=k_{f} k \Delta S_{0}\exp\left[ -k_{rec}(m-1) \tau\right]\frac{1-\left[ \left( 1-k \right)\exp\left( k_{rec}\tau\right) \right]^{m}}{1-(1-k)\exp\left( k_{rec}\tau\right)}$. (SI8.18)

Eq. SI8.18 accounts for the O_3_ generated by fracture in the presence of recombination processes. If friction between surfaces of granite particles is also assumed to generate O_3_, we can expect that the amount of O_3_ generated by friction is proportional to the total available surface area.

After $m$ impacts, the amount of powder never affected by impacts is

$\chi_{0}\left( n \right)=\left( 1-k \right)^{m}$. (SI8.19)

Therefore, the powder affected is

$\chi_{a}\left( m \right)={1-\left( 1-k \right)}^{m}$ (SI8.20)

The total surface area is

$S_{tot}\left( m \right)=\left( 1-k \right)^{m} S_{granite,in}+\left[ 1-\left( 1-k \right)^{m} \right] S_{granite,fin}$, (SI8.21)

which can be also written as

$S_{tot}\left( m \right)=S_{granite,fin}-\left( S_{granite,fin}-S_{granite,in} \right)\left( 1-k \right)^{m}=S_{granite,fin}-\Delta S_{0}\left( 1-k \right)^{m}$. (SI8.22)

In the absence of recombination processes, the total amount of O_3_ generated by friction after $m$ impacts is

$c_{ozone}\left( m \right)=k_{a} \left[ S_{granite,fin}-\Delta S_{0}\left( 1-k \right)^{m} \right]$. (SI8.23)

If we assume that O_3_ starts decaying immediately after its generation with the same recombination rate constant, $k_{rec}$, used for O_3_ generated by fracture, we can write that

$c_{ozone}\left( m \right)=k_{a} \sum_{n=1}^{m} \left[ S_{granite,fin}-\Delta S_{0}\left( 1-k \right)^{n} \right]\exp\left[ -k_{rec}\left( m-n \right) \tau\right]$. (SI8.24)

It follows that

$c_{ozone}\left( m \right)=k_{a}\exp\left( -k_{rec}m \tau\right) \left[ S_{granite,fin}\sum_{n=1}^{m} \exp\left( k_{rec}n \tau\right)-\Delta S_{0}\sum_{n=1}^{m} \left( 1-k \right)^{n}\exp\left( k_{rec}n \tau\right) \right]$, (SI8.25)

Therefore, the total amount of O_3_ generated by friction after $m$ impacts is

$c_{ozone}\left( m \right)=k_{a}\exp\left[ -k_{rec}\left( m-1 \right) \tau\right] \left[ S_{granite,fin}\frac{1-\left[ \exp\left( k_{rec}\tau\right) \right]^{m}}{1-\exp\left( k_{rec}\tau\right)}-\Delta S_{0}\left( 1-k \right)\frac{1-\left[ \left( 1-k \right)\exp\left( k_{rec}\tau\right) \right]^{m}}{1-(1-k)\exp\left( k_{rec}\tau\right)} \right]$. (SI8.26)
